# Whole-genome duplication and hemoglobin differentiation traits between allopatric populations of Brazilian *Odontophrynus americanus* species complex (Amphibia, Anura)^*^

**DOI:** 10.1590/1678-4685-GMB-2017-0260

**Published:** 2019-06-27

**Authors:** Aurora M. Cianciarullo, Claudia R. Bonini-Domingos, Luiz D. Vizotto, Leonardo S. Kobashi, Maria-Luiza Beçak, Willy Beçak

**Affiliations:** 1 Laboratory of Genetics, Instituto Butantan, São Paulo, SP, Brazil; 2 Department of Biology, Laboratory of Hemoglobins and Genetics of the Hematological Diseases, Universidade Estadual Paulista “Julio de Mesquita Filho (UNESP), São José do Rio Preto, SP, Brazil; 3 Department of Zoology, Universidade Estadual Paulista “Julio de Mesquita Filho (UNESP), São José do Rio Preto, SP, Brazil; 4 Laboratory of Ecology and Evolution, Instituto Butantan, São Paulo, SP, Brazil; 5 Universidade Paulista (UNIP) São Paulo, SP, Brazil

**Keywords:** Anura, cryptic species, hemoglobin differentiation, polyploidy, whole-genome duplication

## Abstract

Two allopatric populations of Brazilian diploid and tetraploid *Odontophrynus americanus* species complex, both from São Paulo state, had their blood hemoglobin biochemically analyzed. In addition, these specimens were cytogenetically characterized. Biochemical characterization of hemoglobin expression showed a distinct banding pattern between the allopatric specimens. Besides this, two distinct phenotypes, not linked to ploidy, sex, or age, were observed in adult animals of both populations. Phenotype A exhibits dark-colored body with small papillae, ogival-shaped jaw with reduced interpupillary distance and shorter hind limbs. Phenotype B shows yellowish-colored body with larger papillae, arch-shaped jaw with broader interpupillary distance and longer hind limbs. Intermediate phenotypes were also found. Considering the geographical isolation of both populations, differences in chromosomal secondary constrictions and distinct hemoglobins banding patterns, these data indicate that 2n and 4n populations represent cryptic species in the *O. americanus* species complex. The observed phenotypic diversity can be interpreted as population genetic variability. Eventually future data may indicate a probable beginning of speciation in these Brazilian frogs. Such inter- and intrapopulational differentiation/speciation process indicates that *O. americanus* species complex taxonomy deserves further evaluation by genomics and metabarcoding communities, also considering the pattern of hemoglobin expression, in South American frogs.

## Introduction

After Susumu Ohno’s proposal of evolution by whole-genome duplication (WGD) as a primordial pathway for evolutionary innovations (Ohno, 1970), the speciation process has drawn close attention from evolutionary biologists (Huminiecki and Conant, 2012; Ezaz and Deakin, 2014). The use of Mathematical Physics concepts to predict the development of species allowed for computer simulation, indicating that species differentiation may arise without geographical isolation, in line with the Neutral Theory, which states that species diversity results from random processes acting on similar populations. This method simulates hypothetical evolution process of a virtual population over hundreds of generations (de Aguiar *et al.*, 2009). It is now common sense that genetic differences do not accumulate only when a population is forced into geographical isolation: the emergence of new species can occur over partial insulation only, or even independently of any geographical barriers (Martins *et al.*, 2013). Thus, some traditional organizational principles no longer provide a satisfactory framework, such as the classification of speciation mechanisms by geographical context in allopatric, parapatric and sympatric categories. There is a great debate to promote changes in the classical methodologies about speciation settings and biodiversity (Marie Curie SPECIATION Network *et al.*, 2012; Lemos-Costa *et al.*, 2017).

The fossorial South American frogs of the genus *Odontophrynus* form a distinctive and phylogenetically compact group of warty toad-like burrowers, readily distinguished from other members of the same former *Leptodactylidae* family (Savage and Cei, 1965), which have been recently submitted to phylogenetic analyses that led to a taxonomic revision and consequent reclassification of the genus *Odontoprhynus* as part of the *Odontophrynidae* family (Anura: Neobatrachia) (Pyron and Wiens, 2011). In this genus, only one species - *Odontophrynus americanus* - presents the phenomenon of polyploidy, the first reported occurrence in bisexual natural vertebrate species (Beçak *et al.*, 1966).

At the same year, Sáez and Brum-Zorilla (1966) found chromosome variation in *Odontophrynus americanus* (Amphibia-Anura), which they described as multiple translocations.

Presently, many other distinct bisexual polyploid lineages of amphibians are known, such as the tetraploid, hexaploid, and octaploid frogs, which exist as normally reproducing bisexual population or species (Beçak *et al.*, 1967a, b; Bogart, 1980; Kobel and Pasquier, 1986; Tymowska, 1991). Triploid hybrid specimens (3n=33) were obtained artificially at first by crossing a male *O. cultripes* (2n=22) with a female *O. americanus* (4n=44) (Beçak *et al.*, 1970). The gametes of these 3n (bred for five years in the laboratory) were analyzed. These specimens formed abnormal aneuploid and normal euploid gametes. So although some cells had irregular numbers of chromosomes, others had complete numbers like n, 2n and 3n (Beçak and Beçak, 1970). These gametes could lead to the emergence of higher levels of ploidy, such as hexaploidy (Beçak and Beçak, 1970). Natural triploids and hexaploids were actually discussed in the literature (Batistic *et al.*, 1973; Ruiz *et al.*, 1980, 1981). Gradually, cryptic species or morphologically similar diploid species are being set apart from those they are thought to have evolved from (Beçak *et al.*, 1970; Bogart and Wasserman, 1972; Batistic *et al.*, 1975; Bogart and Tandy, 1976; Mahoney and Robinson, 1980; Tymowska and Fischberg, 1980). More recently, a natural interspecific hybrid between *Odontophrynus cordobae* and *O. occidentalis* specimen belonging to the *Odontophrynus* species complex was documented (Pereyra *et al.*, 2009), sharing exosomatic and chromosomal characteristics of both species. Thus, polyploidy seems to be of considerable evolutionary importance in some vertebrate groups. It is possible that polyploidization occurred frequently during the evolution of higher organisms, with ulterior evolution into a diploid state by chromosomal rearrangement and subsequent diversification of species (Ohno, 1970; Beçak and Beçak, 1974a, 1998; Sparrow and Nauman, 1976; Leipoldt and Schmidtke, 1982; Mable and Bogart, 1995; Beçak and Kobashi, 2004; Huminiecki and Conant, 2012; Beçak, 2014).

Speciation seems to be occurring in our experimental model of the diploid and tetraploid *O. americanus* species complex, not only at the geographical isolation level. Despite their reproductive isolation, diploid and tetraploid specimens collected in the state of São Paulo, Brazil, are morphologically indistinguishable from each other, and also from the holotype deposited at the Museum of Natural History in Paris, which was collected in Buenos Aires, Argentina (Miranda-Ribeiro, 1920). The same is true for the Argentine- Uruguayan populations (Barrio and Pistol de Rubel, 1972). *Odontophrynus lavillai* (Cei, 1985) encompasses diploid populations from Santiago del Estero that were analyzed formerly by Barrio and Pistol de Rubel (1972), with the denomination *O. americanus* referring exclusively to tetraploid specimens (Cei, 1985, corroborated by Rosset *et al.*, 2006, and followed by several authors, as Rocha *et al.*, 2017). Also, *O. cordobae* was described as diploid population (Martino and Sinsch, 2002), followed by *O. maisuma* (Rosset, 2008), and more recently, *O. juquinha* (Rocha *et al.*, 2017).

In the present study we performed a biochemical analysis of the expressed hemoglobins from Brazilian populations of diploid and tetraploid *O. americanus* species complex specimens and we compared them with data found in the literature.

## Materials and Methods

### Specimens

Eight living adult diploid *Odontophrynus americanus* species complex from Botucatu and eight living adult tetraploid *O. americanus* species complex from São Roque - both inner cities of the state of São Paulo, Brazil - weighting 6-18 g, were used.

All guidelines set by the Biodiversity Information and Licensing System / Chico Mendes Institute for Biodiversity Conservation (SISBIO/ICMBio) were complied with. The Biodiversity Committee of the Butantan Institute and the Ministry of Environmental Affairs, through the Brazilian Institute of Environment and Renewable Natural Resources (IBAMA – Process: 02001.005160/2008) have endorsed the present study. Sub-samples used in this study were deposited at the Alphonse Richard Hoge Herpetological Collection - Butantan Institute, in conformity with resolutions of its Genetic Heritage Management Board (CGEN No.147, June 29, 2006. *Odontophrynus americanus* - Registry Number: IBSPCR 0677-0618).

### Karyotyping

Mitotic chromosomes in metaphase were obtained from two animals randomly chosen from the two populations. They were injected subcutaneously with a single dose of 200 μL/10g body weight of 1% colchicine in saline solution. After 2 hours, fragments of intestine of about 1 mm were collected, placed in cold distilled water for 15 min, and fixed for 20 min in a 50% acetic acid solution, before squashing between slide and coverslip. Coverslips were removed in dry ice; the preparations were hydrolyzed for 10 min at 60 °C with HCl 1N, and then stained for 15 min with 2% Giemsa in distilled water, at room temperature (Beçak *et al.*, 1966).

### Cellulose acetate electrophoresis of hemoglobins (Hb)

Ten blood samples of 5 μL were collected from each animal group in 10% EDTA (4:1), hemolyzed in 1% saponin (1:1), homogenized and submitted to cellulose acetate electrophoresis, performed using Tris-EDTA-Borate Buffer (TEB) pH 8.6 at 300 V for 40 min. The bands were stained with 0.5% Ponceau (Naoum, 1990). A 5 μL sample of human blood was collected from a healthy volunteer donor and used as control. Carbonic anhydrase was used as a loading control.

### Isoelectric focusing of Hb

A 0.2% agarose gel was prepared in bi-distilled water, and carrier ampholytes for isoelectric focusing (pH 5-8; 6.5-9 and 3-10) were immediately added. Three fresh 5 μL blood samples from each animal group were hemolyzed by osmotic lysis: cells were rinsed 3 times in 0.8% NaCl, and 3 to 5 volumes of distilled water per volume of rinsed erythrocytes were added. Samples were applied and electrophoresis was performed at 5 mA until samples were transferred to the gel and migration began. The current was then increased to 8 mA for 40 min at 4 °C. After running the gel was fixed for 3 min with 10 mL of 20% Tri-chloride-acetic acid (TCA), and rinsed in distilled water for 2 min. Gels were dehydrated for 12 to 15 h at room temperature, and then stained for 10 to 15 min with 5.5% Ponceau or 0.6% Starch Black (Naoum, 1990).

### Globin chains electrophoresis

Tris-EDTA-borate (TEB)-urea buffer pH 8.6 was prepared and maintained by agitation until use at room temperature. Three samples of 5 μL from each animal group were prepared by addition of 50 μL TEB-urea and 50 μL β-mercaptoethanol to 50 μL hemolyzed blood cells, following incubation for 30 min at room temperature. Then, 6.4 mL of β-mercaptoethanol were added to the TEB-urea running buffer, and the samples were applied exactly at the center of a cellulose acetate sheet. Electrophoresis was performed at 150 V for 80 min. The resulting bands were stained with 0.5% Ponceau or 0.6% Starch Black (Naoum, 1990).

### Agar gel electrophoresis of Hb at acid pH

Agar gels were prepared by adding 200 mg of Bacto-agar (Difco Lab) to 20 mL of phosphate buffer pH 6.2, and phosphate buffer pH 6.2 was used in both electrolytic compartments. Three blood samples of 5 μL from each animal group were applied exactly at the center of the agar gel. Electrophoresis was performed at 100 V for 20 min. The resulting bands were stained with 0.5% Ponceau (Naoum, 1990).

## Results

### Biological characterization

Eight diploid and eight tetraploid *O. americanus* species complex were collected respectively in the surroundings of Botucatu and São Roque cities, state of São Paulo, Brazil. The cities are 154 km apart and there was no overlapping in populations of 2n and 4n animals in both collecting areas. The animals remained burrowed beneath the ground during the day and came to surface at night, to look for food or to mate. Most frogs were captured during the breeding season, which occurs in rainy days of January-March, or sporadically in July-August. Differences in the sound spectrum of mating calls were observed between diploid and tetraploid animals during this period, as previously noted in Argentinian populations (Bogart and Wasserman, 1972; Martino and Sinsch, 2002).

**Figure 1:**
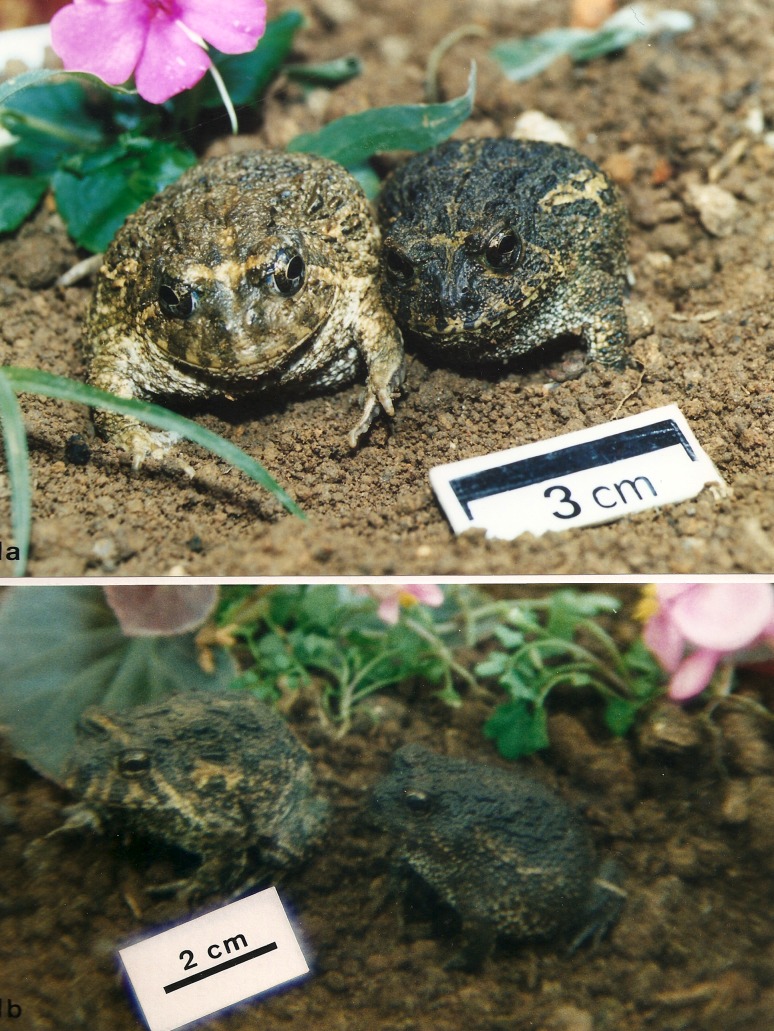
Odontophrynus americanus species complex phenotypes. **(**a) *O. americanus* species complex 4n (left) and 2n (right). Phenotype A is expressed by the tetraploid and phenotype B is expressed by the diploid. (b) *O. americanus* species complex 2n (left) and 4n (right). Phenotype A is expressed by the diploid and phenotype B is expressed by the tetraploid.

Two distinct phenotypes were observed in the adult animals of both populations, whose characteristics were not linked to ploidy, sex or age (Figure 1). Individuals with intermediate phenotypes, but with characteristics of both phenotypes could also be observed. Phenotype A displayed head length almost as large as head width, increased interpupillary distance, light yellowish color and larger papillae on the dorsal skin, intense yellowish colors and lines at the mouth skin around the superior jaw, and longer hind limbs (Figure 1, left side). Phenotype B displayed ogival head, dark color, smaller dorsal skin papillae and shorter hind limbs. Patterns and colors on the mouth skin were usually almost imperceptible (Figure 1, right side).

### Cytogenetic characterization

**Figure 2:**
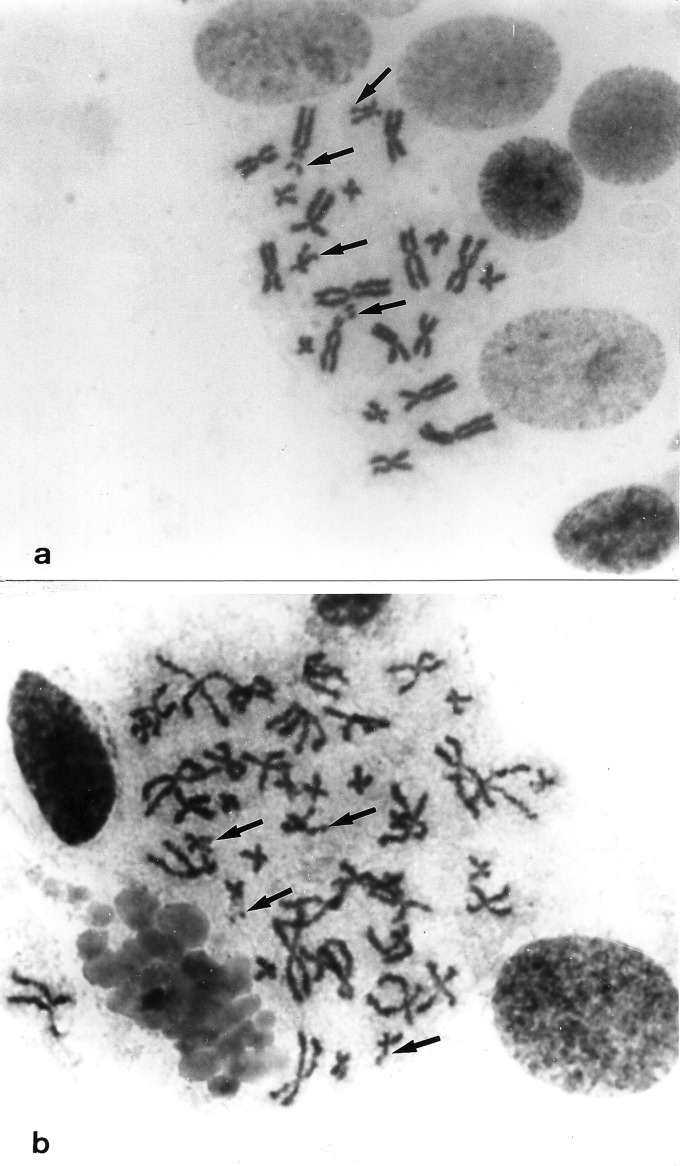
Karyotypes confirming the degree of polyploidy. (a) *O. americanus* species complex 2n = 22 chromosomes, with satellites on pairs 4 and 11 (arrows); (b) *O. americanus* species complex 4n = 44 chromosomes, with satellites on both pairs 11 (arrows).

Karyotypes from two living and recently captured animals were performed, confirming the degree of ploidy according to the locality where the frogs were collected. Diploids *O. americanus* (2n) species complex presented 22 chromosomes, with satellites on pairs 4 and 11*.* Tetraploids *O. americanus* (4n) species complex presented 44 chromosomes, with satellites on both pairs 11 (Figure 2).

### Biochemical characterization

**Figure 3:**
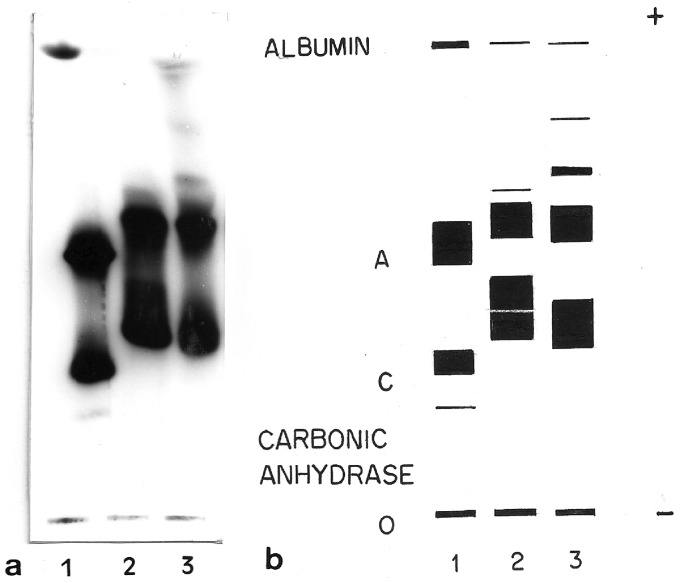
Hemoglobin fractions. (a) Electrophoretic migration pattern of hemoglobin fractions in cellulose acetate. Lane 1: Human hemoglobin AC; lane 2: *O. americanus* species complex 2n; lane 3: *O. americanus* species complex 4n. (b) Schematic representation of the electrophoretic migration pattern.

Blood of living *O. americanus* species complex specimens collected in Botucatu (2n) and São Roque (4n) was used for biochemical assays. Electrophoretic fractionation of Hb, performed in cellulose acetate, revealed four distinct fraction bands in diploid individuals and three bands in tetraploid ones, where the first band apparently consists of two merged fractions (Figure 3).

**Figure 4:**
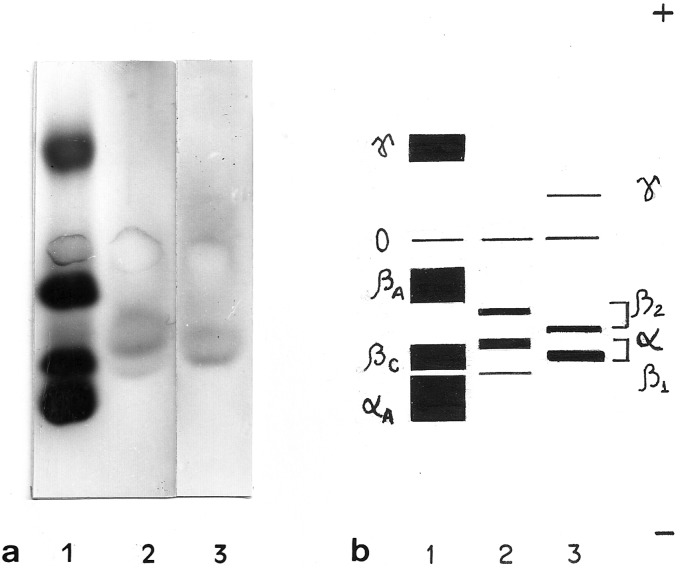
Globin chains. (a) Globin chains electrophoresis performed in cellulose acetate. Lane 1: human hemoglobin AC; lane 2: diploid *O. americanus* species complex; lane 3: tetraploid *O. americanus* species complex*.* Three chains are present in all samples. Human chains correspond to globin αA, βC and βA and γ from the negative pole, respectively. The two main globin chains of the diploid (α-like and β2-like) present a migration pattern that is different from the corresponding main globin chains of the tetraploid. (b) Schematic representation of globin electrophoresis.

In both diploids and tetraploids, two distinct globin types (α-like and β2-like) were detected through globin chain electrophoresis, whose mobility pattern was substantially different from the current in human globin chains (Figure 4). Furthermore, a β1-like chain was present only in diploids, while a *γ*-like chain was present only in tetraploids. These globins also presented a banding pattern which was different from those found in human globin chains.

**Figure 5:**
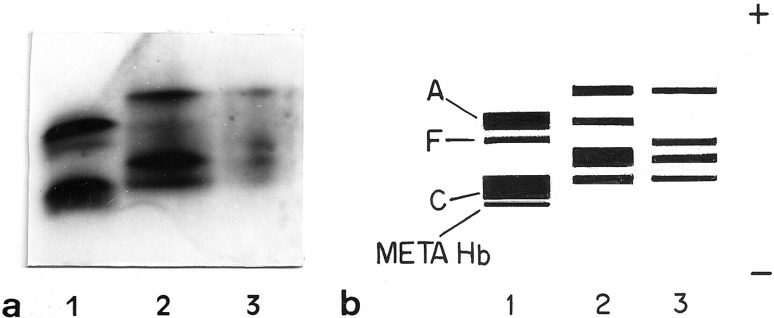
Isoelectric focusing. (a) Migration pattern of hemoglobin fraction in agarose gel. Lane 1: human A, F (fetal), C and Meta Hb; lane 2: diploid *O. americanus* species complex; lane 3: tetraploid *O. americanus* species complex*.* Both amphibians presented four distinct bands. (b) Schematic representation of isoelectric focusing migration patterns.

The electrophoretic pattern of Hb detected by isoelectric focusing showed four distinct fractions or bands in both animals. While electrophoretic mobility was similar between the first and the fourth fractions or bands, it was distinct between the second and third ones (Figure 5).

**Figure 6:**
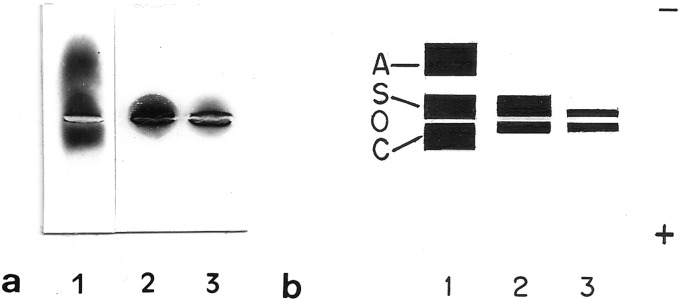
Agar gel electrophoresis (a) Agar gel separation at acid pH electrophoresis did not allow differentiation of hemoglobin types between diploid and tetraploid *O. americanus* species complex*.* Lane 1: human A, S, and C hemoglobin; lane 2: diploid *O. americanus* species complex*.* (b) Schematic representation of acid pH electrophoretic migration patterns.

Acid pH electrophoresis of hemoglobin from both animals showed no differences in the mobility pattern, with absence of polarity in Hb molecules at these experimental conditions. Both samples remained around the application point, indicating absence of variant or mutant hemoglobin besides the main Hb (Figure 6).

## Discussion

The first discovery of animal polyploidy in natural bisexual species of vertebrates was made fifty years ago in the South American frog *Odontophrynus americanus* by Beçak and collaborators (Beçak *et al.*, 1966). Since then, several studies were carried out with polyploid populations of *O. americanus* frogs, but much remains to be done, especially after the advent of new concepts and technologies in molecular biology, which encourages revisiting the past and reorganize the present, preparing for further investigations.

The mapping of the geographical distribution of diploid and tetraploid *O. americanus* in South America showed that diploids tend to live in lower-temperature, above-sea-level regions, from São Paulo to Rio Grande do Sul (Brazil) and in Córdoba (Argentina), whilst tetraploids live in warmer regions of Brazil, ranging from Minas Gerais, São Paulo, and Paraná to Rio Grande do Sul, as well as in Paraguay, Uruguay and Argentina (Beçak and Beçak, 1974b). Tetraploids are thus distributed in a wider area and in several ecological niches when compared to the diploids. Our results showed that diploid and tetraploid populations of the *O. americanus* species complex from the state of São Paulo [Botucatu (804 m above sea level) and São Roque (771 m above sea level) respectively] are allopatric, since both cities are 154 km distant from one another. Karyotypes of recently captured specimens confirmed the diploid and tetraploid conditions, with 22 and 44 chromosomes, respectively. However, sympatry of both diploid and tetraploid specimens of the *O. americanus* species complex had been found in the same pond at different seasons in Pirajuí and Guapiara, both in São Paulo state, Brazil (Ruiz *et al.*, 1981) and at Santa Bárbara do Sul, in Rio Grande do Sul, southern Brazil (Ruiz *et al.*, 1984) and these findings were recently reviewed (Beçak, 2014).

An extensive review about the geographic distribution of diploid and tetraploid populations of the *O. americanus* species complex was done by Rosset *et al.* (2006). The authors found a complex geographic pattern of populations with different ploidy, including areas of syntopy and sympatry, and they also report the occurrence of a naturally triploid specimen and the first record of B-chromosomes in the genus.

Differences in the sound spectrum of the mating calls were observed between the Brazilian diploid and the tetraploid frogs. These differences were recorded in Argentinian frogs (Bogart and Wasserman, 1972; Martino and Sinsch, 2002) and they evince the existence of a prenuptial isolation mechanism that, coupled with genetic incompatibility, are factors that contribute in maintaining both groups apart. Together with other sources of biochemical and genetic evidence it has been proposed that the Argentinian diploid *O. americanus* is a new cryptic species, named *O. cordobae* (Martino and Sinsch, 2002), *O. lavillai* (Cei, 1985), *O maisuma* (Rosset 2008), and *O. juquinha* (Rocha *et al.*, 2017).

Our data demonstrate the occurrence of two phenotypes in both diploid and tetraploid Brazilian frog populations belonging to the *Odontophrynus* species complex, characterizing an intrapopulational polymorphism, which is maintained in sympatry if each population (diploids and tetraploids) is singularly considered, whereas it is in allopatry with regard to each other. An interpopulational difference was also observed in Argentina, in geographically isolated populations of diploid and tetraploid *O. americanus*, although intrapopulational polymorphism has not been detected (Barrio and Pistol de Rubel, 1972). Interpopulational differences were also reported between diploid and tetraploid *O. americanus* from Brazil and Argentina, attributed to the great distance that separates them (Barrio and Pistol de Rubel, 1972). Intra- and inter-specific morphometric variation was found between *Odontophrynus* populations of central Argentina (Grenat *et al.*, 2012). Intrapopulational differences were also found in a tetraploid *O. americanus* population from Rio Cuarto (Córdoba, Argentina) (Barale *et al.*, 1981), with three distinct phenotypes related to dorsal color and skin patterns. Based only on morphological parameters, their three detected phenotypes can be fitted to our phenotypes A and B. A described diploid species from Argentina has been designated as *O. lavillai* (Cei, 1985). Examination of photographic registry suggests that this species corresponds to the diploid and tetraploid *O. americanus* phenotype B described herein, as well as to a distinct diploid *O. americanus* previously described (Barrio and Pistol de Rubel, 1972). The observed phenotypic diversity found in phenotypes A and B can be interpreted as population genetic variability. Maybe in the future these data will be evaluated as indicative of the beginning of speciation in these Brazilian frogs. Therefore, it is clear that further studies with improved molecular biology tools are needed to clarify the correct taxonomic status of the *Odontophrynus* species complex.

The electrophoretic patterns of 6PGD and G6PD enzymes (Schwantes *et al.*, 1969, 1977) revealed identical banding patterns in both 2n and 4n *O. americanus* specimens, which could argue in favor of the hypothesis that the tetraploid *O. americanus* arose by autopolyploidy from the diploid *O. americanus*. On the other hand, there is also the possibility that allopolyploidy has occurred by mating between *O. americanus* 2n and *O. occidentalis* 2n, or their ancestor, which would agree with the electrophoretic patterns of 6PGD and G6PD and with the finding that these two species are sympatric in Oriental Argentina (Barrio, 1964). A third possibility is that other species, now extinct, could have contributed to tetraploidy, since the *Odontophrynidae* family seems to have undergone extensive radiation throughout South America during the Eocene (Pyron and Wiens, 2011), and several geological events such as glaciation, aridness, or others occurred during and after this period, leading to speciation and a great diversity of groups (Savage, 1973).

The biochemical characterization of Hb resulted in distinct banding patterns between both diploid and tetraploid specimens. Variant or mutant Hbs were not detected. Phylogenetic reconstructions should be performed to assess the relative contributions of whole-genome duplication. The information obtained so far in relation to the *in vivo* erythropoiesis in these cryptic Brazilian *O. americanus* populations demonstrates differences at the physiological and molecular levels (Cianciarullo *et al.*, 2000a,b), indicating that a speciation process is occurring and that the cladogenesis should be revised, independently of the morphological variations. New molecular methodologies as combined mitochondrial DNA, nuclear DNA, as well as morphological, genetic, and bioacoustics data should be useful to further characterize these cryptic species.

The most common classification of speciation modes begins with the spatial context in which divergence occurs: sympatric, parapatric, or allopatric. This classification is unsatisfactory because it divides a continuum into discrete categories, concentrating attention on the extremes. It also ignores the fact that speciation is a prolonged process that commonly has phases in different spatial contexts. It has been suggested that it is more productive to study the current balance between local adaptation and gene flow, the interaction between components of reproductive isolation and the genetic basis of differentiation (Butlin *et al.*, 2008).

Regarding the *O. americanus* species complex, some specimens have received local taxonomic status and new nomenclatures over the years, sometimes without a broad community study that encompasses the four South American countries in which their natural habitat is located: Argentina, Brazil, Paraguay and Uruguay (Barrio, 1964; Barrio and Pistol de Rubel, 1972; Barale *et al.*, 1981; Cei, 1985; Martino and Sinsch, 2002; Rosset *et al.*, 2006, 2009; Rocha *et al.*, 2017). A review of the geographic distribution of diploid and tetraploid populations of the *Odontophrynus americanus* species complex indicates an intricate geographic pattern of populations with different ploidy, including areas of syntopy and sympatry (Rosset *et al.*, 2006). The tetraploid *O. americanus* presents three disconnected population groups, which are isolated from one another by diploid populations. One of these tetraploid groups is distributed along central and eastern Argentina, southern Brazil, southern Paraguay, and Uruguay, with the other two population groups inhabiting southeastern Brazil, and northwestern Argentina, respectively. Yet, Rosset *et al.* (2006) included the distribution of both diploid *O. lavillai* and *O. cordobae*, and presented new records of three more allopatric diploid population groups, referred to as *Odontophrynus* sp. Some of these population groups are associated with biogeographic regions. This review indicates that there is a complex distribution pattern of populations of different ploidy, including areas of syntopy and sympatry, and cytogenetic variability. This could indicate the presence of more species, the occurrence of auto- and allopolyploidy, and multiple origins of tetraploidy. Therefore, taxonomic work at the proteomics and genomics levels is needed to resolve this species complex, native from Argentina, Brazil, Paraguay, and Uruguay, and also found in several protected areas (Aquino *et al.*, 2010).

Based on our findings we agree with the proposition made by Rosset *et al.* (2006) and Aquino *et al.* (2010) about the need of a more comprehensive study, which at our point of view ought to be carried out by an international consortium, with the active participation of representative researchers from South American countries, in order to assess the territory inhabited by the herpetofauna of the *Odontophrynidae* family in its maximum extension. Perhaps we shall be surprised by new records and even new species of the genus *Odontophrynus* in areas not previously surveyed, due to their irradiation in search for new habitats.

Barcoding is a DNA-based species identification, which is transforming the traditional approach to the study of biodiversity (Cristescu, 2014). A DNA barcode-based evaluation of the complex genus *Odontophrynus* could greatly contribute for a more precise taxonomic classification. This will be a challenge and incentive to the new generation of biologists, with the perspective on the Earth BioGenome Project (EBP) to sequence, catalog, and characterize the genomes of all of Earth’s eukaryotic biodiversity (Lewin *et al*., 2018).

Assistance from the International Consortium for Zoomorphology Standards (Vogt *et al.*, 2013; Deans *et al.*, 2015) would be of great importance for the reorganization of the South American *Odontophrynidae* family, mainly considering the recent advancements in the computational analysis of phenotypes. Concerning the high number of omics data currently generated, gene ontology is being improved into a more consistent and effective method towards the characterization of functional relationships between existing gene products (Gan *et al.*, 2013; Gan, 2014; Gkoutos *et al.*, 2017).
